# Couple based family planning education: changes in male involvement and contraceptive use among married couples in Jimma Zone, Ethiopia

**DOI:** 10.1186/s12889-015-2057-y

**Published:** 2015-07-21

**Authors:** Tizta Tilahun, Gily Coene, Marleen Temmerman, Olivier Degomme

**Affiliations:** College of Public Health and Medical Sciences, Jimma University, Jimma, Ethiopia; Rhea, Research Center on Gender and Diversity, Brussels University, Brussels, Belgium; International Centre for Reproductive Health, Department of Obstetrics and Gynaecology, Ghent University, Ghent, Belgium

**Keywords:** Couples, Quasi-experimental, Family planning, Educational, Intervention

## Abstract

**Background:**

Family planning contributes substantially in achieving the Millennium Development Goals. Recently, male involvement has gained considerable attention in family planning programs but the implementation thereof remains a challenge. In that context, our study aimed at measuring the effect of a six-month-long family planning education program on male involvement in family planning, as well as on couples’ contraceptive practice.

**Methods:**

We conducted a quasi-experimental research among 811 married couples in Jimma Zone, southwest Ethiopia. Our study consisted of an intervention and a control group for comparative purpose; and surveyed before and after the implementation of the intervention. The intervention consisted of family planning education, given to both men and women at the household level in the intervention arm, in addition to monthly community gatherings. During the intervention period, households in the control group were not subject to particular activities but had access to routine health care services.

**Results:**

We obtained follow-up data from 760 out of 786 (96.7 %) couples who were originally enrolled in the survey. Findings were compared within and between groups before and after intervention surveys. At the baseline, contraceptive use in both control and intervention households were similar. After the intervention, we observed among men in the intervention arm a significantly higher level of willingness to be actively involved in family planning compared to the men in the control arm (p < 0.001). In addition, the difference between spouses that discussed family planning issues was less reported within the control group, both in the case of men and women ((p = 0.031) and (p < 0.001)) respectively. In general, a significant, positive difference in male involvement was observed. Concerning contraceptive use, there was change observed among the intervention group who were not using contraception at baseline.

**Conclusions:**

This study showed that family planning educational intervention, which includes both spouses and promotes spousal communication, might be useful to foster contraceptive practice among couples. The results also offer practical information on the benefits of male involvement in family planning as a best means to increase contraceptive use. Thus, providing opportunities to reinforce family planning education may strengthen the existing family planning service delivery system.

## Background

Worldwide, there is a growing consensus that a good approach to family planning would help in achieving the Millennium Development Goals (MDGs) [1, 2]. Fostering family planning practice alleviates poverty, accelerates socio-economic development, increases child schooling, promotes gender equality, and decreases maternal and infant mortality [3]. In the past five decades, there has been a reproductive revolution in many developing countries, leading to large fertility decreases in Asia, Latin America and North Africa. Sub-Saharan Africa, however, has not experienced the same rapid trend, and today, the region still has total fertility rates (TFR) of around five births per woman [4].

An important cause for these high fertility rates in African countries is the low availability and use of family planning services. The contraceptive prevalence rate (CPR) and unmet need for family planning in 2012 in Sub-Saharan Africa was 25.7 % and 25.1 % respectively, compared to 62.5 % and 12.4 % in all developing countries combined [5]. In that context, Ethiopia ranks as an average Sub-Saharan African country with a CPR of 28.6 % and an unmet need of 25 % of the married women [6]; of these, 16 % have a need for spacing births, and 9 % have a need for limiting [6]. Estimates suggest that reducing this unmet need for family planning might lower the Ethiopian TFR by as much as 37.5 % from an actual 4.8 to a desired three children per woman [6]. To achieve the above-mentioned target, there is a need to have an integrated approach in family planning programs. For that matter, targeted interventions and programs on family planning are increasingly shifting from the individual factor approach to the multi-directional approach, in favour of models that examine the intersection of factors across individual, social, and cultural domains [7]. Particularly in the context of developing countries, the decision regarding family planning is not solely made by couples but also by the husband´s family and other relatives. The extended family system, typical for many developing countries, and wide social networks have a major influence on a couples’ contraceptive practice. To address these complex and manifold aspects that determine contraceptive use, the socio-ecological model framework is often used in family planning research [8]. Within this paper, we have used this framework to study individual, relational, community, and societal factors that influence a ‘couple’s contraceptive practice' [9].

A factor that has recently gained attention as an important determinant of contraceptive use is the role of the husband in the family planning decision-making process. According to Allen et al. (2014), men’s role in family planning involves making the decision on contraceptive practice [10], but in less-developed countries, findings indicate that male participation is less common [11, 12]. Previous research recommended that men should also be involved in family planning programs [12] but in most countries worldwide, these programs have so far focused exclusively on women as a target group [13, 14]. Some studies have shown that intensive efforts to reach the targeted family planning coverage are most successful when they involve men as well, not only women [15]. Research in Malawi suggested that targeting men for family planning interventions may significantly increase contraceptive uptake [16]. A study of family planning services at the Ethiopian town, Wolaita, also recommended that information, education, and communication be provided to change the behaviour of men regarding contraceptive practice [14]. Others suggested that consistent and regular family planning education for both spouses, might be more effective than targeting one specific gender only [17]. The importance of involving husbands in family planning programs has also been documented for increasing the use of modern contraceptives instead of traditional methods [18]. However, research on couples' use of contraception mostly focused on the knowledge, attitudes, discussion, and intentions regarding family planning rather than on the actual impact of programs on contraceptive use and use of family planning services [19]. In brief, to achieve higher levels of contraceptive prevalence, efforts need to be done to encourage spousal communication and agreement, and to stimulate men’s participation in family planning [15]. To date, too little research has been conducted to identify the best ways to achieve this [20, 21].

Considering both the research results mentioned above and the high fertility rate in Ethiopia, the purpose of this study in Jimma Zone, Ethiopia, was to measure the effect on a couple’s contraceptive use of a family planning intervention that encouraged spousal communication. In addition, we studied changes in male involvement in family planning among those exposed to the intervention and in a control group. As a whole, this paper reports the implementation of the intervention and a comparative analysis before and after the intervention among the two groups.

## Methods

### Study setting

The study was conducted in Jimma Zone, one of 14 administrative zones of the Oromia region in the southwest of Ethiopia. Its capital, Jimma, is situated 352 km to the southwest of the national capital, Addis Ababa. Jimma Zone has 17 districts and one special zone. Based on the 2007 national census, it has a population of about 2.5 million, with a little more than half being men [22]. The rural part accounts for 89.5 % of the total population size of the zone, in which the dominant ethnic group is the Oromo. An estimated 52 % of Jimma Zone's residents have access to health posts, which is the lowest level of the primary healthcare system (Jimma Zone administration: Public Relations and Information Office, unpublished document). The healthcare delivery within the zone is carried out through 13 health centres, 26 health posts, 65 health stations, and 2 hospitals (Jimma Zone administration: Public Relations and Information Office, unpublished document). Among these, 35 are privately owned and non-governmental organization clinics.

### Sampling

A multi-stage sampling design was used with districts as primary sampling units (PSU), and sub-districts (kebeles) as secondary sampling units (SSU). The study covered three districts i.e. Seka, Manna and Gomma, in which six kebeles were randomly selected: Goyoo qechema, Koffie, Gobiemuleta, Haro, Gembie and Bulbulo. In each selected kebele, a complete census of married couples was compiled to be used as sampling frame. Married couples were then randomly sampled from each locality, based on a computer generated random number list until the required size was achieved. From the three selected districts, two sub-districts were randomly taken and assigned to either a control or intervention group. The sample size was computed using Minitab version 14 statistical software in order to detect a 10 % or more decrease in an unmet need for family planning. With an alpha of 0.05 and 80 % power, the minimum required sample size was estimated at 388. By adding 10 per cent to account for non-responses, the final sample size was 427 couples. Villages included in the study were selected according to their geographical proximity in order to minimize geographical variations because each chosen village needed to be situated at a similar distance to a health facility. Considering the large distances between villages, cross-contamination was not likely.

We designed our study using a quasi-experimental design to assess the impact of an educational campaign intervention. A total of 854 married men and their wives (427 in control and 427 in intervention group) were selected using systematic random sampling. To be eligible, couples had to meet the following criteria: i) legally married men and their wives; ii) living together in the same house during the six months prior to the baseline data collection in the study area; iii) planning to stay in the area for one year starting from the time of data collection; iv) the wife's age was between 15 and 49 years; and v) the wife was not pregnant at the moment of the baseline data collection. Husbands within a polygamous marriage (who had more than one wife) were excluded from the analysis to decrease redundancy of information. Fieldwork took place from March 2010 to May 2010 (the baseline household survey) and from March 2011 to April 2011 (the follow-up survey) in the same three intervention sub-districts and three control sub-districts. The survey instruments were developed from a validated questionnaire and were considered valid and reliable as it was tested and used by other studies to obtain information on couples about knowledge, attitude and contraceptive practice [23–26]. A three-day training was given to the interviewers. After house numbers were assigned within each sub-district, random house numbers were generated using SPSS® version 16 for Windows®. Households were listed prior to the selection of eligible respondents. In total, 1,622 individuals (811 couples) were interviewed for the baseline study. The same approach was used to collect the follow-up household survey data. A total of 1,546 individuals were interviewed for the follow-up study (drop-out of 4.7 %). There was little variation in the participants due to the marginal dropout rate during follow-up.

### Measurement

The researchers prepared a semi-structured questionnaire for the quantitative method. Data were collected in the local language with separate questionnaires sharing a similar core set of questions for men and women; informed consent was received before participation. The pre- and post- intervention data collection was conducted after six months of intervention with similar questionnaires. Couples who participated in the two rounds of interviews were included in the analysis. After the last instruction on family planning education and community gathering, we submitted the same questionnaire to the men and their wives. The intervention program for married groups described in this article incorporated both men and women. It was carried out by male and female community agents who used different communication materials (flyers, reading materials and leaflets).

### Intervention

After the baseline survey conducted in early 2010, family planning education was given in villages located in the sub-districts assigned to the intervention arm. The intervention consisted of 1) family planning education on different methods of contraception through flyers, booklets, and face-to-face discussions, and 2) promotion of husband-wife discussions on family planning. Family planning education was given to both men and women at the household level in the intervention arm, in addition to monthly community gatherings. A health officer trained the three male and three female community agents who were hired for this study’s purpose and undertook the intervention activity. These community agents had completed high school, whereas the health officer held a degree; they all spoke the local language fluently. The community agents were involved in the community under study through family planning interventions. They were chosen to be community agents based on this extensive experience. They were assigned to provide information on different methods of contraception by several means of communication (flyers, booklets, and face-to-face discussions) in order to promote husband-wife discussion on family planning and to teach reproductive health rights to couples focusing on family planning. Some of the types of contraceptives discussed during intervention were pills, injectables, implants, condoms, and standard days method. The condom, for example, was discussed among the male contraceptive methods. The intervention was designed and executed in partnership with local leaders. During the routine visits to each household, the coordinators performed random spot checks to see how the topic was covered. In addition, the principal investigator supervised the proper implementation of activities throughout the intervention period. During this period also, the normal health care routine was carried out for both the control and intervention group, but there were no initiatives taken by other health care providers. There were some efforts delivered by mass media such as television and radio, but these were not considered as confounding factors because both the control and intervention groups were also exposed to the other citizens of the country.

### Data analysis

We assessed the effect of the intervention on the use of contraception, the involvement of men in family planning, and spousal discussions on family planning issues. The dependent variables were contraceptive use among couples and the male involvement in family planning. We calculated crude odds ratios (ORs) as well as adjusted them (aORs) for survey design effects using conditional logistic regression methods. We used generalized linear models that accounted for stratification, clustering and weights (svyglm in R ‘survey’ package). Since quasi-experimental research can be subject to biases and can be confounding due to unforeseen baseline differences between the intervention and control group, we used an Inverse Probability of Treatment Weighted (IPTW) analysis to account for these differences by using propensity scores that were estimated through multivariate logistic regression. Variables that were included in this regression were the literacy of both spouses, whether or not family planning had been discussed previously, and baseline attitudes of the husband towards being involved in the couple’s family planning practices. As a result, our findings are adjusted for potential baseline differences in those variables. We used for all statistical analysis STATA ® version 10 for Windows® and R version 3.0.1.

### Ethical considerations

Ethical clearance of the study was obtained from the research and ethics committee of the College of Public Health and Medical Sciences, Jimma University, Southwest Ethiopia and Ghent University’s Ethical Committee in Belgium. Written consent was obtained from each man and woman participating in the study after the data collectors explained the purpose of the study using a predefined information sheet. Written informed consent was taken from spouses on the behalf of those wives who were less than 18 years old, considering the cultural context of the study area. No compensation was rendered as a direct incentive to the participants. The ethics committees approved this consent procedure.

## Results

During the baseline survey we achieved a response rate of 92 % in our overall sample. For a detailed analysis of this survey, we refer to a previously published article [27]. Post-intervention, 96.7 % of the baseline study participants responded to our survey, including the intervention arm and control group. A slight difference was found between the intervention and control group with respect to those who dropped out during the follow-up.

### Contraceptive use

After the intervention, 45.5 % of the couples reported using a form of contraception. No statistically significant difference in contraceptive use was found between the intervention (47.6 %) and control (43.4 %) arms. The contraceptive use in the control group was the same at baseline and post-intervention (43.4 %), while in the intervention group, we noticed an increase from 41.9 % to 47.6 %. The levels of contraceptive use in the surveyed couples after the intervention are presented in Table 1. Among those who were using contraceptives at baseline, approximately one quarter was no longer using any form of contraception at the post-intervention survey; this was the case in both arms. We noted a larger decrease in contraceptive use in the intervention group compared to the control group, although this was not statistically significantly larger. Among those who were not using contraceptives at baseline, we found a positive association between the intervention and use of contraception after the intervention: in the intervention group 28.6 % had started using contraceptives compared to 17.2 % in the control group. (aOR = 1.90; p = 0.014) (see Table 1). When stratifying this group according to reasons given at baseline for not using contraception, the effect of the intervention remained significant only among those who were not using contraception at baseline due to a lack of adequate knowledge on the topic (aOR = 2.77; p = 0.034); in all other cases, the effect was no longer significant, possibly due to small sample sizes.

**Table 1:** Contraceptive use by couples Jimma zone, Ethiopia, 2014

	*Total*	Control group	Intervention group	*Odds ratio*	
				*crude*	*adjusted*
At baseline	*328/772 (42.5 %)*	164/379 (43.3 %)	164/393 (41.7 %)	*0.94 (p = 0.665)*	*0.98 (p = 0.905)*
At follow-up	*351/771 (45.5 %)*	165/380 (43.4 %)	186/391 (47.6 %)	*1.18 (p = 0.248)*	*1.25 (p = 0.159)*
Contraceptive use at follow-up according to use of contraception at baseline					
Users at baseline	*248/328 (75.6 %)*	127/164 (77.4 %)	121/164 (73.8 %)	*0.82 (p = 0.441)*	*0.92 (p = 0.772)*
Non-users at baseline^a^	*102/442 (23.1 %)*	37/215 (17.2 %)	65/227 (28.6 %)	*1.93 (p = 0.005)*	*1.90 (p = 0.014)*
- no need	*59/225 (26.2 %)*	23/105 (21.9 %)	36/120 (30 %)	*1.53 (p = 0.168)*	*1.14 (p = 0.680)*
- not willing or not allowed	*3/26 (11.5 %)*	1/16 (6.2 %)	2/10 (20 %)	*3.75 (p = 0.286)*	*2.30 (p = 0.528)*
- lack of knowledge	*26/109 (23.9 %)*	10/69 (14.5 %)	16/40 (40 %)	*3.93 (p = 0.003)*	*2.77 (p = 0.034)*
- too expensive	*3/5 (60 %)*	1/2 (50 %)	2/3 (66.7 %)	*2 (p = 0.709)*	*1.30 (p = 0.888)*

^a^Reasons for not using contraceptives at baseline labeled as “no need” include wanting another child, recently married, and recently given birth; “not willing or not allowed” include being against family planning, not allowed by the spouse, not allowed by the family, and not allowed by the religion; “lack of knowledge” include not knowing methods of contraception, fear of side effects, and not knowing where to get contraception. Only 303 out of 442 couples that were not using any form of contraception at baseline reported one or more reasons for this. Categories are not mutually exclusive

### Spousal discussions on family planning issues

Overall, respondents from the intervention arm reported higher levels of spousal discussion on family planning than those from the control arm. Spousal discussion of family planning issues in the past were reported by approximately four out of five spouses in the intervention arm. In the control arm, this ranged from 43.2 % as reported by wives to 68.9 % as reported by the husbands. In both cases, the crude difference between intervention and control arm was significant (among women: OR = 4.7; p < 0.001 and among men: OR = 1.77; p = 0.001); after adjustment for baseline differences, the difference in what the men reported was borderline significant (aOR = 1.36; p = 0.068) (see Table 2). Compared to the couples in the control group, we observed more couples in the intervention arm who said they discussed family planning more frequently than at baseline. This difference is more important among women (aOR = 7.80; p < 0.001) than among men (aOR = 1.41; p = 0.031). Finally, there were significantly fewer spouses in the intervention couples that stated women could decide about family planning without consent of her husband (aOR = 0.06; p < 0.001). The intervention-control difference was equally present when the respondents were asked about the frequency of these discussions: in the control group, about 30 % reported discussing family planning often or very often with their partner, compared with more than twice that percentage in the intervention arm (among men: aOR = 4.7; p < 0.001 and among women: aOR = 4.46; p < 0.001). When comparing the post-intervention data to the baseline data, both men and women in the intervention group reported a higher increase in discussions on family planning than those in the control group. This difference was particularly significant among the wives. Finally, we found a significant difference between the two groups with respect to spousal consensus on family planning decisions: respondents from the intervention group were much less likely to agree with the fact that women can decide on family planning alone without the consent of their husbands (10.2 % and 15.4 % in the intervention group versus 19 % and 63.1 % in the control group).

**Table 2:** Post-intervention spousal discussions on family planning issues, Jimma zone, Ethiopia, 2014

	*Total*	Control group	Intervention group	*Odds ratio*	
				*crude*	*adjusted*
Percentage of couples that discussed family planning issues					
*as reported by the husband*	*574/772 (74.4 %)*	261/379 (68.9 %)	313/393 (79.6 %)	*1.77 (p = 0.001)*	*1.36 (p = 0.068)*
*as reported by the wife*	*471/773 (60.9 %)*	164/380 (43.2 %)	307/393 (78.1 %)	*4.7 (p < 0.001)*	*3.69 (p < 0.001)*
Percentage of couples that discussed family planning issues that discussed it often or very often					
*as reported by the husband*	*309/574 (53.8 %)*	82/261 (31.4 %)	227/313 (72.5 %)	*5.76 (p < 0.001)*	*4.73 (p < 0.001)*
*as reported by the wife*	*272/471 (57.7 %)*	53/164 (32.3 %)	219/307 (71.3 %)	*5.21 (p < 0.001)*	*4.46 (p < 0.001)*
Percentage of couples that reported an increase in discussing family planning issues compared to the baseline survey					
*as reported by the husband*	*246/771 (31.9 %)*	104/379 (27.4 %)	142/392 (36.2 %)	*1.50 (p = 0.009)*	*1.41 (p = 0.031)*
*as reported by the wife*	*302/772 (39.1 %)*	64/379 (16.9 %)	238/393 (60.6 %)	*7.56 (p < 0.001)*	*7.80 (p < 0.001)*
Percentage of individuals who agreed women can decide about family planning without consent of her husband					
*among husbands*	*112/771 (14.5 %)*	72/378 (19 %)	40/393 (10.2 %)	*0.48 (p = 0.001)*	*0.56 (p = 0.007)*
*among wives*	*289/742 (38.9 %)*	231/366 (63.1 %)	58/376 (15.4 %)	*0.11 (p < 0.001)*	*0.10 (p < 0.001)*
*both husband and wife*	*58/740 (7.8 %)*	55/364 (15.1 %)	3/376 (0.8 %)	*0.05 (p < 0.001)*	*0.06 (p < 0.001)*

### Men’s involvement regarding family planning

Based on the response from the husbands, our results suggest higher levels of involvement of the husbands in the intervention group for each of the three assessed indicators. Table 3 shows that in general, approximately half of the male respondents stated they had the intention to go with their spouses to facilities offering family planning services and to cover the costs for these services. Similarly, 28.8 % of the husbands responded they were willing to be actively involved in family planning, with significantly more willing men in the intervention group as compared to the control group (OR = 93.4; p < 0.001) (see Table 3). Since baseline differences regarding these variables existed between the intervention and control group, we analysed the data separately, according to the baseline answers that were given. The findings remained statistically significant after these adjustments, with larger odds ratios among those men who had answered negatively at baseline. We also asked women who were using contraception about the actual involvement of their husband in family planning. The data supports the findings observed among the husbands’ responses, albeit less significantly. Moreover, significant differences were found among those couples in which the husband was already actively involved at baseline; however, among those where no husband involvement was reported at baseline, no significant difference was found. This is possibly due to small sample sizes.

**Table 3:** Post-intervention willingness among men to be involved in family planning Jimma zone, Ethiopia, 2014

	*Total*	Control group	Intervention group	*Odds ratio*	
				*crude*	*Adjusted*
Willingness as reported by the husband					
Go together to FP service	*376/773 (48.6 %)*	73/380 (19.2 %)	303/393 (77.1 %)	*14.2 (p < 0.001)*	*10.7 (p < 0.001)*
- among those already willing at baseline	*309/452 (68.4 %)*	*55/181 (30.4 %)*	*254/271 (93.7 %)*	*34.2 (p < 0.001)*	*25.3 (p < 0.001)*
- among those not willing at baseline	*66/320 (20.6 %)*	*17/198 (8.6 %)*	*49/122 (40.2 %)*	*7.15 (p < 0.001)*	*5.71 (p < 0.001)*
Finance costs	*341/773 (44.1 %)*	66/380 (17.4 %)	275/393 (70 %)	*11.1 (p < 0.001)*	*8.28 (p < 0.001)*
- among those already willing at baseline	*289/438 (66 %)*	*51/169 (30.2 %)*	*238/269 (88.5 %)*	*17.8 (p < 0.001)*	*13.9 (p < 0.001)*
- among those not willing at baseline	*51/334 (15.3 %)*	*14/210 (6.7 %)*	*37/124 (29.8 %)*	*5.95 (p < 0.001)*	*4.82 (p < 0.001)*
Get FP together	*223/773 (28.8 %)*	5/380 (1.3 %)	218/393 (55.5 %)	*93.4 (p < 0.001)*	*80.6 (p < 0.001)*
- among those already willing at baseline	*194/306 (63.4 %)*	*1/82 (1.2 %)*	*193/224 (86.2 %)*	*504 (p < 0.001)*	*460 (p < 0.001)*
- among those not willing at baseline	*28/465 (6 %)*	*4/297 (1.3 %)*	*24/168 (14.3 %)*	*12.2 (p < 0.001)*	*10.3 (p < 0.001)*
Actual involvement as reported by the wife (only couples using a form of contraception)^a^					
Go together to FP service	*299/349 (85.7 %)*	128/163 (78.5 %)	171/186 (91.9 %)	*3.12 (p < 0.001)*	*3.06 (p < 0.001)*
- where the husband was already involved at baseline	*180/199 (90.5 %)*	*80/93 (86 %)*	*100/106 (94.3 %)*	*2.71 (p = 0.046)*	*3.26 (p = 0.024)*
- where the husband was not involved at baseline	*29/50 (58 %)*	*17/33 (51.5 %)*	*12/17 (70.6 %)*	*2.26 (p = 0.196)*	*2.31 (p = 0.193)*
Finance costs	*288/349 (82.5 %)*	121/163 (74.2 %)	167/186 (89.8 %)	*3.05 (p < 0.001)*	*3.10 (p < 0.001)*
- where the husband was already involved at baseline	*166/199 (83.4 %)*	*58/81 (71.6 %)*	*108/118 (91.5 %)*	*4.28 (p < 0.001)*	*4.10 (p = 0.001)*
- where the husband was not involved at baseline	*37/50 (74 %)*	*33/45 (73.3 %)*	*4/5 (80 %)*	*1.45 (p = 0.747)*	*1.01 (p = 0.993)*
Get FP together	*256/349 (73.4 %)*	94/163 (57.7 %)	162/186 (87.1 %)	*4.95 (p < 0.001)*	*4.70 (p < 0.001)*
- where the husband was already involved at baseline	*120/139 (86.3 %)*	*28/40 (70 %)*	*92/99 (92.9 %)*	*5.63 (p = 0.001)*	*6.09 (p = 0.001)*
- where the husband was not involved at baseline	*59/110 (53.6 %)*	*42/86 (48.8 %)*	*17/24 (70.8 %)*	*2.54 (p = 0.056)*	*2.45 (p = 0.078)*

^a^Only 249 out of 349 women who answered these questions during the follow up interview also answered them at baseline. As a result, the disaggregated results are only based on a subset consisting of these 249

## Discussion and conclusions

Our study showed higher follow-up levels of contraceptive use among those couples who were not using contraception at baseline that participated in our family planning intervention compared to couples in the control group. In particular, those couples who reported lack of knowledge as the principle reason for not using contraception seemed to have benefited the most from this intervention. This is not surprising, as the intervention mainly focused on awareness and on being involved in family planning education. Similar to other research findings, our study also showed an association between an increase in contraceptive use and increased spousal discussion [28].

Judged by the findings analysed for this study, three main outcomes were noted as the indicators of male involvement in family planning. Firstly, the intervention led to an increase in the men's intention to go to family planning services with their spouses, even if differences observed at the baseline among control and intervention were negligible. Secondly, when male involvement in family planning was measured from the women’s perspective, it appeared that husbands of women who were already contraceptive users were more involved. Third, agreement in reporting spousal communication as well as male involvement was better among the intervention group. These findings can be interpreted in two ways, depending on the context. One possible explanation could be that it was the positive influence of the added information by the intervention program that triggered men to accompany their spouses who were already attending family planning services. Another explanation could be that male involvement in family planning can possibly only be high for users since with non-users, male involvement is obviously not an issue.

Our findings suggest that overall, men responded that they discussed family planning issues within the couple slightly more often than their counterparts. However, we found this discrepancy was only present among the control group. These results indicate that couples’ education could help to decrease the discord between spouses about family planning by creating common understanding, which, in turn, has a positive effect on the couples' contraceptive practice. This finding is in agreement with a Nigerian study, which elaborated the advantage of giving family planning education for both men and women to foster contraceptive practice [19]. The frequency of discussion was also higher among the intervention group. with the similarity in reporting between spouses. Consequently, we can say that the more both spouses were informed, the more the couple’s attitude towards family planning had changed.

Given the above findings, an unusual result noted in the intervention group was that many spouses responded that they could not take decisions about family planning without the consent of their husbands. This suggests that either the spouses prefer to take the decision jointly, or that – since the intervention is conducted for a short period only – the spouses think that the cultural belief of their husbands who are the decision makers in family planning at the household, does not allow for a behavioural change. As such, the short duration of the intervention could be a limitation. It is also notable that researchers found that, in Nigeria and Bangladesh, husbands´ responses predicted a couple’s subsequent use of family planning [19, 29]. Furthermore, this study revealed the need to promote a high involvement of men in family planning programs in order to revive marital dynamics. However, our findings also suggest that in order to bring about behavioural change, an intensive and protracted intervention program should be implemented.

There are several study limitations that should be acknowledged. First, we relied on self-reported measures of behaviour. It is important to note that semi-structured questionnaires were used to assess the effect of family planning education using pre- and post- intervention survey. Second, the follow-up and family planning education were restricted to six months. Lastly, as we carried out a community-based study, it was difficult to control all factors for contraceptive practice because a community-based intervention study can be influenced by several economic and socio-political factors. Despite its limitations, this study provides preliminary data that could apply to family planning education that focuses on both men and women, and it could be taken as a lesson learned. Moreover, a large sample size was employed, which generated great confidence in the results. In addition, educational materials and health messages were delivered during community gatherings in the presence of men and women together. The result of this analysis indicates that family planning education had a significant impact on spousal discussion about family planning and on male involvement in family planning.

As seen from this study, adopting family planning education that incorporates gender level counselling may improve contraceptive practice. Moreover, offering brief follow-up sessions to the couples and promoting spousal discussion about family planning may increase contraceptive prevalence. Yet, this family planning education intervention may be more promising and effective if it would be implemented for a longer time and if cultural beliefs would be considered in detail.
